# The distinction between irritability and anger and their associations with impulsivity and subjective wellbeing

**DOI:** 10.1038/s41598-023-37557-4

**Published:** 2023-06-27

**Authors:** Maria Gröndal, Karl Ask, Stefan Winblad

**Affiliations:** Department of Psychology, University of Gothenburg, Box 100, 405 30 Gothenburg, Sweden

**Keywords:** Psychology, Human behaviour

## Abstract

Irritability, anger, and impulsivity have important associations with psychological well-being. However, studying the internal relationships between such emotional constructs is challenging, largely because of the lack of precise operational definitions and extensively validated measurement tools. The aim of this study was to examine relationships between the above emotional constructs and how they relate to satisfaction with life and perceived negative impact on different life domains. Participants (*N* = 471) completed a self-report questionnaire online. Correlational analyses showed that higher levels of irritability and trait anger were associated with lower life satisfaction. Impulsivity displayed complex relationships with life satisfaction, with some aspects (sensation seeking) showing a positive relationship and others (urgency, lack of perseverance) showing a negative relationship. A two-factor Confirmatory Factor Analysis treating irritability and anger as separate constructs showed a better fit compared with a one-factor model, indicating that irritability and anger should be treated as separate constructs. An exploratory moderation analysis showed that higher irritability predicted increased anger only for participants scoring average to high on urgency (a facet of impulsivity). Our findings increase the understanding of the relationship between these dispositional constructs and supports the conceptualization of irritability and anger as related but distinct constructs.

## Introduction

Emotional constructs are often “fuzzy” by nature and tend to be challenging to operationalize^1,2^. Therefore, it is crucial to continually evaluate measures of related emotional constructs to investigate their degree of conceptual overlap and unique relationships with meaningful real-life outcomes. Irritability and anger are two closely related constructs frequently occurring in both healthy individuals and those with a pathological condition. From a research and clinical point of view, however, they are poorly understood and often improperly used interchangeably^3,4^. Using validated self-report measurements in a non-clinical sample, the current study aims to increase knowledge about the conceptual overlap and separation between irritability and anger, their relationships with variables pertaining to impulsivity, as well as the emotional variables’ associations with two subjective indicators of real-life outcomes—satisfaction with life and perceived negative impact on different life domains.

### Irritability and anger

Broadly defined, subjective experience of irritability refers to an excessive sensitivity to sensory stimuli, with a lowered threshold for responding to the stimuli with anger or aggressive behavior^4–9^. In contrast to the related emotion anger, feelings of irritability can occur with seemingly no clearly identified trigger or antecedent^4,10^ and are instead often associated to physiological/biological deficiencies, such as experience of stress, inadequate sleep, physical pain, or low blood sugar^4^.

Feeling angry is usually defined as an emotional state that involves displeasure of varied intensity, from mild annoyance to intense fury^11^. Compared with irritability, anger is a more scientifically established construct, although defining it as a single psychobiologically distinct phenomenon has proven difficult^12,13^. Anger is generally triggered by the experience of frustrating situations (e.g., goal hindrance) or by being personally insulted (for instance treated unfairly, blamed or unjustified)^14^. The closest related behavioral response to anger is aggression, generally defined as behaviors directed towards another individual or object where the immediate intention is to cause harm^14^. A person with high trait anger interprets many situations as annoying or frustrating and, to a high extent, reacts to the situations with elevated anger^11^. Anger is, in other words, generally regarded as more closely related to aggressive behavior than is irritability^15^.

Since irritability can develop without outward expressions or symptoms^16^, it is beneficial to measure it at an experience-based level, such as with self-reported measurement tools. However, a major limitation of previous research is that irritability has predominantly been assessed using self-reported single-item measures or measures of related constructs such as anger or aggressiveness^4^. Of the currently existing irritability scales, the Brief Irritability Test (BITe) developed by Holtzman et al.^8^ has succeeded best at measuring irritability with minimal overlap with related constructs and focusing on the inner experiences instead of outward responses^4^. While the authors of the BITe claim that the scale aims to capture the frequency of *state* irritability during the last 2 weeks, they express a belief that irritability has both state and trait properties^8^. To our knowledge, however, the BITe has not yet been validated as a trait measure. In contrast, among self-report measures of anger, a distinction between current experienced state and a general trait tendency exists. One of the most widely used scale to measure anger is the State-Trait Anger Expression Inventory-2 (STAXI-2)^11^, which not only captures the state and trait components of anger, but also the tendencies to control and express anger.

The incidence of irritability and anger is also substantial in clinical contexts, as evidenced by the inclusion of irritability in 15 and of anger in 10 psychiatric disorders in the DSM-V, including mood disorders, trauma- and stress-related disorders, disruptive, impulse control and behavioral disorders, substance-related and addictive disorders, and personality disorders^17^. A noteworthy differentiation between these constructs lies in the fact that irritability is a feature of both internalized states, such as anxiety and depression, and externalized states, such as acting out, impulsivity, or rebelliousness. In contrast, anger symptoms are predominantly observed in diagnoses associated with externalized states. Nonetheless, this differentiation is not absolute, as anger and irritability may co-occur in some conditions. The high prevalence of irritability challenges previous conceptualizations that characterized irritability as a milder form of anger^11^ Given its widespread manifestation across DSM-V categories and its association with significant distress^15^, such an approach is untenable. However, there is a lack of consensus in clinical settings regarding the difference between irritability and anger^4^.

Although it should be clear from this section that irritability and anger are closely related, knowledge of the specific features that distinguish the two has not been established. In the current study we therefore tested psychometrically how irritability and anger differ from each other by conducting a Confirmatory Factor Analysis (CFA). Further, measures addressing the emotional process, specifically, *when*, *how*, and *to what extent* emotional experiences are regulated, could provide additional important clues of how the constructs differences^18^. To deepen the understanding of possible regulation differences (in addition to measures of anger regulation tendencies) four facets of impulsivity were examined.

### Impulsivity

Impulsivity is an umbrella term describing various rapid, under-regulated behavioral reactions to internal or external stimuli, coupled with little forethought to possible negative consequences of such reactions^19^. Overall, researchers agree that there are several underlying mechanisms to impulsive behaviors^20^ and that impulsivity should be regarded as a multidimensional construct^21^. Studying how impulsivity is related to emotional constructs can generate a deeper understanding of how the emotional experience is regulated (or dysregulated)^22^, and it is evident that the tendency to act on impulses influences how we express and experience emotions^23^.

Some facets of trait impulsivity have been described as predictors of aggression; that is, behavior derived from anger experiences^19,24^. However, the specific ways in which impulsivity relates to and interacts with irritability has, to our knowledge, not previously been explored. In the current study, we used an impulsivity measure (UPPS Impulsive Behavior Scale)^23^ that includes four distinct psychological processes that lead to impulsive behaviors; urgency, sensation seeking, (lack of) premeditation, and (lack of) perseverance. *Urgency* refers to the tendency to experience strong impulses, mostly under conditions of negative affect. During the experience of negative emotions, strong feelings of urgency are likely to facilitate impulsive behaviors which may alleviate the negative emotions in the short term but may have harmful long-term consequences. Previous studies have linked urgency with aggressive behavior and violence^25^. Additionally, urgency is defined as emotional impulsivity and is shared among different psychological disorders, including borderline personality disorder, eating disorders, and depression^25–27^. *Sensation seeking* refers to an individual’s openness to exploring dangerous, exciting activities and has been associated with both negative outcomes (such as substance use)^28^ and positive outcomes (such as psychological resilience)^29^. (Lack of)* perseverance* refers to the inability to stay on task despite boredom and is related to attention problems such as in Attention Deficit Hyperactivity Disorders (ADHD)^30^. Lastly, (lack of) *premeditation* refers to the tendency not to reflect or deliberate on the consequences of behaviors before engaging in them, which, for instance, has been associated with antisocial personality disorder^31^ and violence^32^.

### The current study

The current study had two main goals. First, to explore the relationships between irritability, anger, and impulsivity, which have not previously been investigated in conjunction. To this end, we first tested how the constructs of irritability and anger differed from each other psychometrically by conducting a CFA. Moreover, we explored whether impulsivity is a moderator of the relationship between irritability and anger.

The second goal of the study was based on hypotheses formulated in the preregistration prior to the data collection. The aim of these hypotheses was to evaluate the relationships between affective disposition variables—irritability, anger, and impulsivity—and subjective indicators of real-life outcomes—satisfaction with life and perceived negative impact on different life domains. The following hypotheses was formulated:

H1: Satisfaction with life will correlate negatively with (a) irritability, (b) trait anger, (c) impulsivity.

H2: Self-rated negative impact on different life domains will correlate positively with (a) irritability, (b) trait anger, and (c) impulsivity.

## Method

### Participants and statistical power

A total of 576 individuals completed the full questionnaire, of whom 105 (18.2%) were excluded from all data analyses (see “Exclusion criteria”). The final sample included 471 individuals recruited from the research participant pool at the Department of Psychology, University of Gothenburg, from a national online platform for research participant recruitment (https://www.studentkaninen.se/) and through announcements at a public library. The average age of the participants was 33.16 years (*SD* = 11.27, *Mdn* = 31, min = 18, max = 75), of which 355 (75.4%) were women, 112 (23.8%) were men, and 4 (0.8%) identified with other genders. In terms of current occupation, 372 participants (79.0%) reported they were employees or students, 52 (11.0%) unemployed, 25 (5.3%) were on parental or medical leave, 10 (2.1%) were retired, and 12 (2.5%) “other”. Participants had an average of *M* = 15.23 years of education (*SD* = 3.29, *Mdn* = 15).

A sensitivity analysis conducted in G*Power^33^ indicated that our final sample size (*N* = 471) offered 80% power to detect a bivariate correlation of *r* = 0.128 (α = 0.05).

### Procedure and materials

Data were collected between June 19 and September 1, 2020. The study was conducted online, and each participant provided informed consent before completing the online questionnaire. Participants received 40 SEK (≈ 4 EUR) for their participation, which lasted on average 24 min (*SD* = 19 min). The preregistration for the study is available at [https://osf.io/f9hg2/]. The research was carried out in accordance with the guidelines for good research practice issued by the Swedish Research Council^34^. The Swedish Act Concerning the Ethical Review of Research Involving Humans (SFS 2008:192) regulates the types of research involving humans that shall undergo ethics review. Because the current research did not fulfil any of the conditions that necessitate review^34^^,p.30^, it did not undergo formal ethics review. Specifically, no information that would be considered sensitive personal data was collected in our survey. Nonetheless, the study was carefully planned in collaboration with a representative of the Swedish Ethical Review Authority to conform with internationally accepted standards for research ethics^35^.

### Measures

The full questionnaire and a more detailed description of each instrument used in this study can be found on the study’s project page on OSF.

#### Brief Irritability Test (BITe)

The BITe^8^ has 5 items on which participants rate the frequency of experienced irritability (e.g., “I have been grumpy”) in the last 2 weeks on a six-point scale (1 = *never,* 6 = *always*). The internal consistency of the scale in the current sample was ω_*total*_ = 0.91.

#### State-Trait Anger Expression Inventory-2 (STAXI-2)

The STAXI-2^11^ was used to measure various aspects of anger. STAXI-2 includes 57 items using four-point rating scales (1 = *not at all/almost never*, 4 = *very much/almost always*). The *state anger* subscale includes 15 items (e.g., “I feel angry”) and measures the current experience of anger (ordinal ω_total_ = 0.97)^36^. The *trait anger* subscale measures the general disposition to experience anger with 10 items (e.g., “I am quick tempered”; ordinal ω_total_ = 0.89). The remaining items comprise four major components consisting of eight items each: *Anger expression-out* (AX-O; e.g., “I express my anger”) measures the tendency to outwardly express anger towards other people or objects (ordinal ω_total_ = 0.81); *anger expression-in* (AX-I; e.g., “I keep things in”) measures the tendency to direct feelings of anger inward (ordinal ω_total_ = 0.82); *anger control-out* (AC-O; e.g., “I control my temper”) measures the ability to control/suppress angry feelings (ordinal ω_total_ = 0.76); and *anger control-in* (AC-I; e.g., “I take a deep breath and relax”) measures the tendency to control angry feelings by cooling off when angered (ordinal ω_total_ = 0.88).

#### UPPS Impulsive Behavior Scale (UPPS)

The UPPS^23^ includes 45 statements measuring four different facets of trait impulsivity. The scale uses a four-point response scale (1 = *agree strongly*, 4 = *disagree strongly*) measuring the following impulsivity traits: (lack of) *premeditation* (e.g., “I am a cautious person”; ordinal ω_total_ = 0.88), (lack of) *perseverance* (e.g., “I finish what I start”; ordinal ω_total_ = 0.85), and *sensation seeking* (e.g., “I’ll try anything once”; ordinal ω_total_ = 0.89), *urgency* (e.g., “I have trouble controlling my impulses”; ordinal ω_total_ = 0.92). In the English version of the UPPS, urgency is referred to as *negative urgency*.

#### Satisfaction With Life Scale (SWLS)

The SWLS, developed by Diener et al.^37^, was used as a measure of subjective well-being with five statements (e.g., “In most ways my life is close to my ideal”). Participants rated their agreement on a seven-point scale (1 = *strongly disagree*, 7 = *strongly agree*). In the current sample, the internal consistency of the items was high (ω_total_ = 0.91).

#### Negative impact on different life domains

Directly after completing each of the instruments of irritability, anger, and impulsivity, participants were asked to rate the extent to which the respective trait has a negative impact on their (a) work/studies, (b) free-time activities, and (c) social relations. The items were rated on a six-point scale (1 = *never*, 6 = *always*). This measure was constructed specifically for the purpose of this study and reflects the diagnosis criteria of life domains negatively affected by specific symptoms of different conditions in the DSM-V^17^.

#### Attention checks

On the page directly following each of the instruments of irritability, anger, and impulsivity, participants were presented five options and asked to select the most suitable option that described the questions on the previous page. This measure was constructed specifically for the purpose of this study to exclude participants who failed to pay attention to the question content.

### Exclusion criteria

The following exclusion criteria were formulated prior data collection; participants were excluded from all data analyses (a) if they had completed the questionnaire in less than 5 min (*n* = 0) and/or (b) if they failed to correctly answer any of the attention checks (*n* = 87). Additionally, participants who participated more than once (*n* = 16) or were under 18 years of age (*n* = 2) were also excluded from all data analyses. A total of 105 participants were excluded.

## Results

### Confirmatory analyses

Table 1 presents descriptive statistics and bivariate correlations for the scales measuring irritability (BITe), anger (STAXI-2), impulsivity (UPPS), and satisfaction with life (SWLS). In support of our hypotheses, the total score on SWLS correlated negatively with the total score of BITe (H1a), STAXI-2 trait anger (H1b), UPPS (lack of) perseverance and urgency (H1c). All correlations were significant at *p* < 0.001. However, failing to support H1c, two subscales in UPPS ((lack of) premeditation and sensation seeking) did not correlate negatively with SWLS, where sensation seeking instead showed a slight positive correlation.

**Table 1:** Observed correlations between emotional measurement scales and satisfaction with life.

	1	2	3	4	5	6	7	8	9	10	11	12	13
1. BITe	–												
2. STAXI state	0.375***	–											
3. STAXI trait	0.456***	0.442***	–										
4. STAXI A-CI	0.010	− 0.022	− 0.038	–									
5. STAXI A-CO	− 0.211***	− 0.134**	− 0.399***	0.585***	–								
6. STAXI A-XI	0.435***	0.269***	0.397***	0.206***	− 0.026	–							
7. STAXI A-XO	0.314***	0.285***	0.661***	− 0.048	− 0.400***	0.354***	–						
8. STAXI AXindex	0.359***	0.266***	0.539***	− 0.640***	− 0.785***	0.458***	0.629***	–					
9. UPPS S	0.029	0.023	0.128**	0.044	0.083	0.019	0.038	− 0.031	–				
10. UPPS PM	0.055	0.068	0.222***	− 0.152***	− 0.242***	− 0.082	0.191***	0.185***	0.257***	–			
11. UPPS PS	0.217***	0.209***	0.191***	− 0.096	− 0.220***	0.280***	0.208***	0.315***	− 0.060	0.218***	–		
12. UPPS U	0.481***	0.303***	0.614***	0.000	− 0.350***	0.464***	0.476***	0.475***	0.120**	0.338***	0.400***	–	
13. SWLS	− 0.301***	− 0.193***	− 0.210***	0.084	0.203***	− 0.361***	− 0.215***	− 0.341***	0.130**	0.000	− 0.436***	− 0.419***	–
*M*	13.91	17.79	15.43	21.44	25.04	17.96	12.14	31.97	31.19	22.62	20.35	27.85	20.54
*SD*	4.85	5.56	4.67	5.45	3.74	4.75	3.27	10.65	7.96	5.94	5.4	8.06	7.33
Range	24	39	24	24	19	24	17	74	34	31	28	35	30

*BITe* Brief Irritability Test, *STAXI state* STAXI-2 state subscale, *STAXI trait* STAXI-2 trait subscale, *STAXI AC-I* STAXI-2 anger control-in subscale, *STAXI AC-O* STAXI-2 anger control-out, *STAXI AX-I* STAXI-2 anger expression-in, *STAXI AX-O* STAXI-2 anger expression-out, *STAXI AX-index* STAXI-2 anger expression index, *UPPS S* UPPS sensation seeking subscale, *UPPS PM* UPPS (lack of) premeditation subscale, *UPPS PS* UPPS (lack of) perseverance subscale, *UPPS U* UPPS urgency subscale, *SWLS* Satisfaction with Life Scale. *p* < 0.05, **p < 0.01, ***p < 0.001. Significant values are in bold.

Taken together, these results show, as predicted, that higher levels of irritability and trait anger are associated with lower perceived satisfaction with life. However, the relationships between impulsivity and satisfaction with life were less straightforward than anticipated. While individuals high in urgency and lack of perseverance did report lower satisfaction with life as predicted, lack of premeditation was unrelated to satisfaction with life and sensation seeking showed a slight positive correlation.

The upper panel of Table 2 provides bivariate correlations between the measures of irritability, trait anger, and impulsivity (rows), and the self-rated extent to which those traits impact negatively on three areas of life: work/studies, free time activities, and social relations (columns). In line with our hypotheses, most of these correlations were positive and significant, indicating that individuals with heightened levels of irritability (H2a), trait anger (H2b), and impulsivity (H2c) are more likely to perceive these traits as barriers to everyday functioning. The only exception to the above pattern was the sensation seeking subscale of UPPS, for which correlations with impact on life were either weak (work/studies) or non-significant (free time activities and social relations). Thus, H2c received only partial support. In addition, compared with anger, irritability was perceived as significantly more often having a negative impact on all three rated life domains (work/studies: *t*(908.27) = − 4.96,* p* < 0.001, *d* = − 0.32, 95% CI [− 0.45, − 0.19]; free time activities: *t*(915.77) = − 5.51, *p* < 0.001, *d* = − 0.36, 95% CI [− 0.49, − 0.23]; social relations: *t*(933.81) = − 4.89, *p* < 0.001, *d* = − 0.32, 95% CI [− 0.45, − 0.19]). Further analyses on mean differences of the self-reported negative impacts between the measured dispositional variables can be found at https://osf.io/f9hg2/.

**Table 2:** Correlations between irritability, anger, and impulsivity (rows) and self-reported negative impact of the constructs on different life domains (columns).

	Work/studies	Free time activities	Social relations
BITe	0.431***	0.426***	0.518***
STAXI trait	0.315***	0.410***	0.443***
UPPS S	0.119**	0.045	0.042
UPPS PM	0.259***	0.157***	0.140**
UPPS PS	0.497***	0.415***	0.329***
UPPS U	0.445***	0.447***	0.535***
Irritability impact *M* (*SD*)	2.23 (1.12)	2.20 (1.10)	2.56 (1.14)
Anger impact *M* (*SD*)	1.90 (0.92)	1.83 (0.94)	2.21 (1.05)
Impulsivity impact *M* (*SD*)	2.37 (1.21)	2.29 (1.16)	2.38 (1.14)

*BITe* Brief Irritability Test, *STAXI trait* STAXI-2 trait subscale, *UPPS S* UPPS sensation seeking subscale, *UPPS PM* UPPS (lack of) premeditation subscale, *UPPS PS* UPPS (lack of) perseverance subscale, *UPPS U* UPPS urgency subscale. **p < 0.05, ***p < 0.01. Significant values are in bold.

### Preliminary exploratory analyses

To explore the appropriateness of treating irritability and anger as separate constructs, two CFAs were conducted. To handle the fact that BITe and STAXI-2 contained different scale steps, Maximum Likelihood with Robust standard errors (MLR) was used as an estimator in both models. The first model tested the variables in the BITe and STAXI-2 trait as a one-factor model, indicating an inadequate fit (RMSEA = 0.174; SRMR = 0.133; CFI = 0.619; TCI = 0.556). The second model was a two-factor model where the BITe and STAXI-2 trait were separated, indicating a comparatively better fit (RMSEA = 0.100; SRMR = 0.098; CFI = 0.876; TCI = 0.853). To identify possible disturbances in the two-factor model, we studied the modification index and found that two items in the STAXI-2 trait greatly affected the model's fit. These two items turned out to be very similar and was about feeling angry when you felt that those around you did not appreciate what you had done (e.g., work tasks). By allowing these two variables to correlate freely in the model, we were able to achieve a good fit (RMSEA = 0.079; SRMR = 0.063; CFI = 0.909; TCI = 0.924). The obvious difference between the one-factor model and the two-factor model supports the assumption that irritability and anger measure different concepts. For further details on these analyses see OSF https://osf.io/f9hg2/.

### Exploratory moderation analyses

Based on the relatively strong correlations between irritability (BITe), trait anger (STAXI-2), and urgency (UPPS), we were interested in examining if different facets of impulsivity moderate the relationship between irritability and trait anger. We examined this potential interaction in a multiple linear regression model with irritability as the focal predictor variable, the impulsivity subscales (sensation seeking, premeditation, perseverance, urgency) as proposed moderators, and trait anger as the outcome variable. As shown in Table 3, a significant interaction effect was observed, such that the association between irritability and anger was moderated by the impulsivity subscale urgency.

**Table 3:** Results of regression model predicting anger from irritability and impulsivity.

	Unstandardized estimate *b* [95% CI]	Standardized estimate β [95% CI]	*p*
BITe	− 0.365 [− 0.809, 0.079]	− 0.333 [− 0.739, 0.072]	0.107
UPPS S	0.047 [− 0.086, 0.181]	0.095 [− 0.172, 0.361]	0.486
UPPS PM	− 0.071 [− 0.261, 0.119]	− 0.103 [− 0.378, 0.172]	0.462
UPPS PS	− 0.087 [− 0.297, 0.122]	− 0.114 [− 0.388, 0.160]	0.413
UPPS U	0.091 [− 0.043, 0.225]	0.163 [− 0.078, 0.404]	0.184
BITe * UPPS S	− 0.001 [− 0.010, 0.008]	− 0.033 [− 0.295, 0.228]	0.801
BITe * UPPS PM	0.007 [− 0.006, 0.020]	0.149 [− 0.127, 0.426]	0.288
BITe * UPPS PS	0.002 [− 0.012, 0.016]	0.036 [− 0.229, 0.301]	0.788
BITe * UPPS U	0.014 [0.006, 0.024]	0.393 [0.158, 0.629]	0.001

*R*^2^ = 0.44.

**Figure 1:**
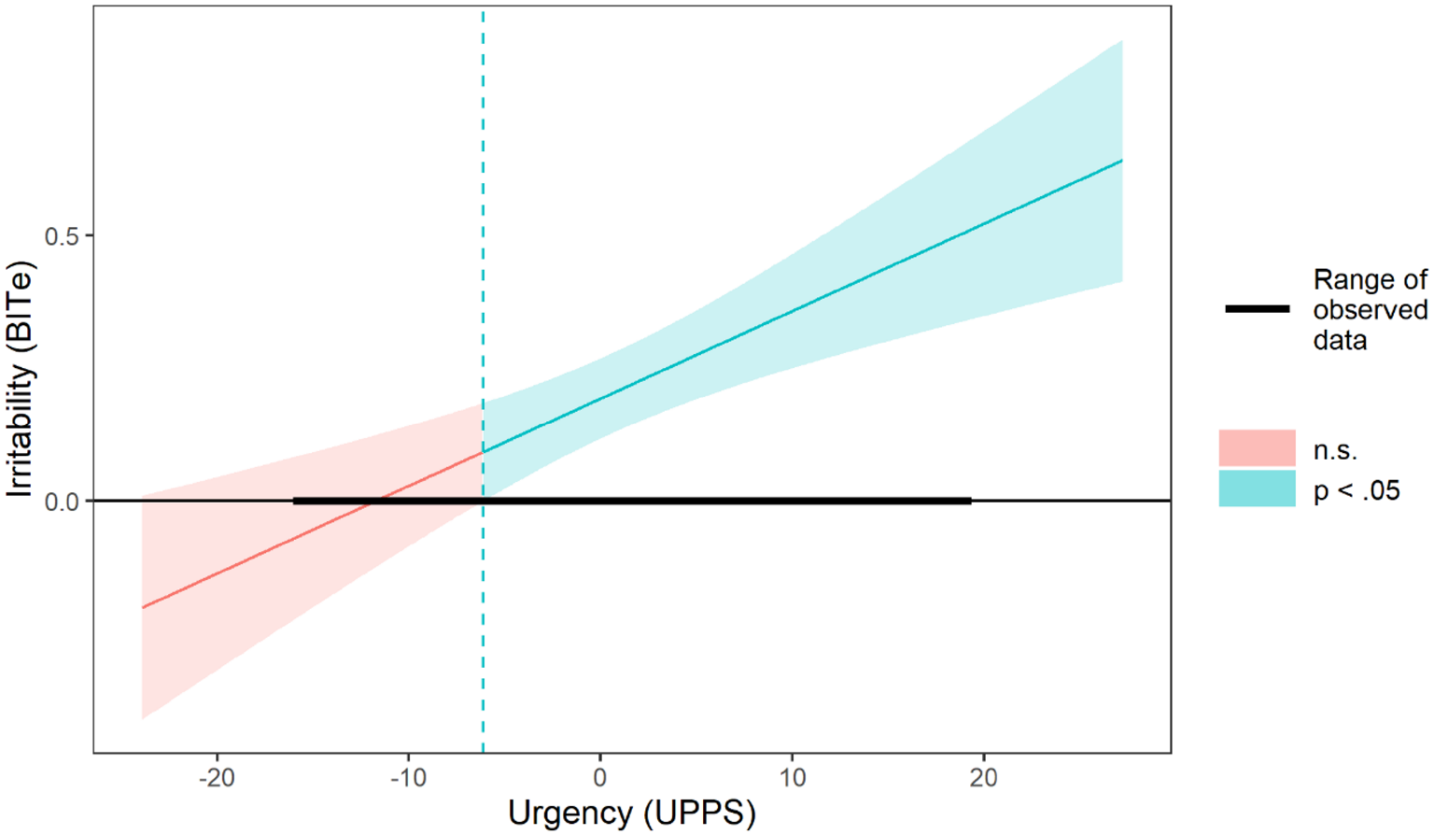
Conditional effect plot for the association between trait irritability and trait anger as a function of trait urgency. Note. Urgency scores have been mean centered on this plot. The shaded regions represent a 95% confidence interval. The y-axis represents the strength and direction of the association between irritability and anger.

A conditional effect analysis using the Johnson-Neyman technique showed that the coefficient for irritability was significant and positive at urgency values equal to or higher than 6.12 points below the sample mean (see Fig. 1). This result indicates that higher irritability levels predicted increased anger only for respondents scoring average to high on the impulsivity urgency subscale.

## Discussion

The results indicated that higher levels of irritability and trait anger were associated with lower satisfaction with life and higher perceived negative impacts on the life domains work/studies, free time activities, and social relations. The negative consequences of the experience of irritability and anger were expected, and their negative impact on people’s general life highlights the importance of understanding the complexity of these constructs. Further, the results showed that participants found that their irritability had a more negative life impact than anger, possibly because experiences of irritability tend to be more frequent than experiences of anger^38^.

Regarding impulsivity, we found distinct patterns across the four facets of the impulsivity scale, which supports the notion that impulsivity is best conceptualized as a multidimensional construct^21^. The subscales urgency and (lack of) perseverance were related to lower satisfaction with life and negative impact on different life domains, which was in line with our hypothesis and previous findings. However, a positive relationship between sensation seeking and satisfaction with life was found in our sample. This may reflect that, in a non-clinical population, sensation-seeking represents adaptive and appetitive personality traits^39^, whereas its maladaptive role has previously been documented in clinical populations^30,40^. (Lack of) premeditation was not associated with satisfaction with life but was related to negative perceived impact on primarily work/studies. Again, the distinction between clinical and non-clinical samples might be relevant for the interpretation of these findings. (Lack of) premeditation involves acting in the moment without regard to consequences^23^, but at moderate levels, found among non-clinical individuals, its negative consequences may be limited.

When irritability and anger were examined psychometrically through the exploratory CFA, the two constructs appeared to be distinct from each other. However, this result should be interpreted with some caution because irritability measured with BITe is a more state-oriented construct while anger as measured with STAXI-2 looks at behavioral patterns of trait characteristics. The result may therefore to some extent be due to a difference between state and trait conditions. Nevertheless, as irritability and anger have historically been regarded as interchangeable constructs in scientific and clinical contexts^4^, their distinct properties remain largely unexplored. Therefore, to further develop the field we encourage future studies to include separate measures for the two constructs. Our findings indicate that each individual construct can have unique explanatory value, which can deepen the understanding of, for example, how emotional reactions in psychiatric conditions can be expressed differently.

Our exploratory moderation analysis showed that high levels of irritability predicted increased anger for participants with average to high levels of the impulsivity facet urgency. This result provides a key to understanding the conditions under which irritability is likely (or unlikely) to translate into expressions of anger. One possible interpretation of the finding is that urgency plays an important role in regulating the threshold between irritability and anger. Specifically, higher levels of urgency may facilitate the transformation of the inner experience of irritability into an outward expression of anger. Another possible interpretation of the exploratory finding is that individuals with both high trait anger and urgency have a generally higher tendency to feel irritable.

The correlational nature of our study limits the possibilities to draw any certain causal conclusions from the exploratory results discussed above. However, we argue that our results are consistent with the causal direction supported by previous knowledge about the constructs. First, irritability is described as a mood with a lowered threshold for expressing anger^4^, and it could thus be argued that irritability comes before anger and not the opposite. Second, urgency is the tendency to be impulsive when experiencing negative feelings^23^. Hence, in this context, urgency can be regarded as a reaction to, rather than an antecedent of, the negative experience of irritation. This finding could be of potential clinical importance. When working with emotion regulation in a therapeutic context, it is important to understand the antecedents of an emotion^41^ and the underlying cognitive processes generating the emotion^42^. If irritability leads to anger because of a dysregulation of urgent impulses, it would be important to focus on the regulation of impulsivity as well as explore potential situations that irritate the client. An argument for especially focusing on irritability as a target for clinical intervention is that it is more likely to be a state condition that fluctuates under external and internal influences. Previous research show that emotional impulsivity (specifically urgency) is a common trait among individuals with personality disorders and that the degree of impulsivity is positively associated with the severity of the condition^27^. Therefore, when combined with irritability, it may create a volatile combination. This raises an intriguing question regarding the extent to which individuals can learn to manage their level of irritability, a question worth addressing in future clinical therapeutic trials. However, before it is suitable to test this in clinical interventions, the speculation regarding the underlying mechanism of the irritability–anger pathway should be addressed in future studies using confirmatory and/or longitudinal designs.

## Conclusion

The current research has shown that irritability and anger are related but distinct constructs, which are both negatively correlated with life satisfaction. In the long term, a refined conceptual understanding of these dispositions has the potential to inform clinical interventions for the treatment of emotional impulsivity. Furthermore, the present study advances the field by suggesting that urgency—a facet of impulsivity—may regulate the threshold at which internally experienced irritability translates into outwardly directed anger.

## Data Availability

The datasets analyzed during the current study are publicly available at OSF [https://osf.io/f9hg2/]. For further questions regarding the datasets analyzed please contact the corresponding author.
